# Drought-Tolerance of Wheat Improved by Rhizosphere Bacteria from Harsh Environments: Enhanced Biomass Production and Reduced Emissions of Stress Volatiles

**DOI:** 10.1371/journal.pone.0096086

**Published:** 2014-05-08

**Authors:** Salme Timmusk, Islam A. Abd El-Daim, Lucian Copolovici, Triin Tanilas, Astrid Kännaste, Lawrence Behers, Eviatar Nevo, Gulaim Seisenbaeva, Elna Stenström, Ülo Niinemets

**Affiliations:** 1 Dept. of Forest Mycology and Plant Pathology, Uppsala BioCenter, SLU, Uppsala, Sweden; 2 Dept. of Plant Physiology, Estonian University of Life Sciences, Tartu, Estonia; 3 Institute of Evolution, The University of Haifa, Haifa, Israel; 4 Dept. of Chemistry and Biotechnology, Uppsala BioCenter, SLU, Uppsala, Sweden; 5 Estonian Academy of Sciences, Tallinn, Estonia; University of Delhi South Campus, India

## Abstract

Water is the key resource limiting world agricultural production. Although an impressive number of research reports have been published on plant drought tolerance enhancement via genetic modifications during the last few years, progress has been slower than expected. We suggest a feasible alternative strategy by application of rhizospheric bacteria coevolved with plant roots in harsh environments over millions of years, and harboring adaptive traits improving plant fitness under biotic and abiotic stresses. We show the effect of bacterial priming on wheat drought stress tolerance enhancement, resulting in up to 78% greater plant biomass and five-fold higher survivorship under severe drought. We monitored emissions of seven stress-related volatiles from bacterially-primed drought-stressed wheat seedlings, and demonstrated that three of these volatiles are likely promising candidates for a rapid non-invasive technique to assess crop drought stress and its mitigation in early phases of stress development. We conclude that gauging stress by elicited volatiles provides an effectual platform for rapid screening of potent bacterial strains and that priming with isolates of rhizospheric bacteria from harsh environments is a promising, novel way to improve plant water use efficiency. These new advancements importantly contribute towards solving food security issues in changing climates.

## Introduction

The world population is predicted to increase beyond 8 billion by 2030 implying major challenges for agricultural sector to secure food availability [1]. A key challenge for plant growth is global water shortage, limiting crop yields already today in more than 70% of arable lands, and the drought limitations further gain in importance in the near future as agricultural activities expand to less fertile areas to satisfy growing demands for food [2], [3]. Accordingly, understanding and improving plant survival and growth under restricted water availability is of central significance in contemporary plant science.

A variety of strategies has been used to improve the drought tolerance of crops, including traditional selection methods and genetic engineering [4]. Drought stress tolerance is a complex trait, and although a large number of genes involved in plant drought stress responses has been identified, large gaps still remain [4]–[6]. Finding of suitable phenotypes is further complicated by exposure of plants to multiple environmental stressors in the field either simultaneously or sequentially. Although a certain overlap may occur in plant physiological responses to different stressors, plant responses to each stress alone or in combination with other stresses can be different [7]. Furthermore, plant response to drought stress is also developmentally regulated [8]–[10].

Given the strongly varied environments with complex stress environment, ontogenetic variations, large number of different crops with huge variety of cultivars, and the plethora of genes expression of which need to be altered or novel genes engineered into plants, it is currently unclear whether the engineering technology will develop fast enough to cope with rapidly increasing food demands in near future [11]. As a relatively simple and low-cost alternative strategy, the use of free-living plant growth-promoting bacteria (PGPB) has been highlighted as a promising broad-spectrum means to improve plant growth [11]–[14]. The PGPB ability to increase plant growth and provide protection against various pathogens has been frequently reported and applied since 50ies in agricultural systems. The very first report on PGPB-induced drought stress tolerance was published by Timmusk and Wagner only in 1999 [14], [15]. From the huge variety of potential microorganisms, gram-positive rhizosphere bacteria are most likely to be commercially applied in wide range of areas of limited water resources due to the ease of their handling and endospore-forming ability that facilitate efficient colonization under environmental stress conditions [11], [14], [16]–[19].

Although the exact mechanisms of plant drought stress tolerance enhancement by rhizosphere bacteria remain largely speculative, possible explanations include: (1) production of hormones like abscisic acid, gibberellic acid, cytokinins, and auxin; (2) production of essential enzymes, 1-aminocyclopropane-1-carboxylate (ACC) deaminase to reduce the level of ethylene in the root of developing plants; (3) induced systemic resistance by bacterially-produced compounds; (4) formation of bacterial biofilm i.e. extracellular matrix [12], [13], [20], [21] [17]. In particular, an extracellular matrix formed by bacterial biofilm can provide almost infinite range of macromolecules beneficial for plant development and growth. Biofilms contain sugars and oligo- and polysaccharides that can play various roles in bacteria-plant interactions, e.g., in improving water availability in root medium. The water retention capacity of some polysaccharides can exceed several-fold their mass [17]. In fact, it has been demonstrated that even a small polysaccharide alginate content in the biofilm facilitates maintenance of hydrated microenvironment [22].

Here we studied the importance of habitat of rhizospheric bacteria in plant drought stress tolerance enhancement. The bacteria included in this study were isolated from harsh environments such as South Facing Slope (SFS) in ‘Evolution Canyon’ (EC), Israel, Mt. Lemmon, Arizona, USA and Tina plain, Giza, Egypt as described by us earlier [23]. We showed that the bacterial capacities to solubilize phosphorus, form biofilms, tolerate high salt concentrations and the bacterial activity of ACC deaminase were significantly higher in stressed environments [23]. The bacteria have coevolved with their hosts over long periods of time, and thus, they are expected to control to a large extent plant adaptation to their belowground environment. Furthermore, bacteria intimately associated with host plant roots have likely contributed to plant adaptation to harsh environmental conditions at evolutionary time-scales as well [23].

Higher plants respond to water shortage by multiple physiological and morphological modifications [19], [24]–[26]. Characteristic drought responses include stomatal closure, osmotic adjustment by increased content of cellular osmotica, changes in foliage structure and total whole plant leaf area and root system characteristics, collectively improving water conservation, water use efficiency (rate of production per water use) and water extraction from drying soil [24]–[28]. Despite adaptive modifications, reactive oxygen species (ROS) and accumulation of free radicals associated with damage to membranes and buildup of lipid peroxides can occur in severely water-stressed plants [29]–[31]. While changes in ROS formation after reaching the threshold water stress level may initially activate host defense mechanisms, their continued production under drought stress can be the ultimate cause of damage in water-stressed plants, indicating that the balance between formation and detoxification of activated oxygen species is critical for cell survival. Induced changes in different ROS-scavenging enzymes and enhanced photosynthetic efficiency have been reported in PGPB-inoculated plants, helping the plants to cope with drought stress [32], [33]. Some of key ROS-scavenging enzymes include glutathione-ascorbate cycle enzymes glutathione reductase (GR) (EC 1.8.1.7) and monodehydroascorbate reductase (MDHAR) (EC 1.6.5.4) [34]. Superoxide dismutase (SOD) (EC 1.15.1.1) and catalase (CAT) (EC 1.11.1.6) act as main enzymatic scavengers for superoxide (O_2_^−^) and hydrogen peroxide (H_2_O_2_) [33].

In plant responses to a multitude of stresses, induction of volatiles plays a key role [35], [36]. The stress-induced volatiles serve as signals for development of priming and systemic responses within the same and in neighboring plants [37]–[39], and the emission rates of volatiles are generally strongly associated with the severity of stress [40]–[44]. Thus, monitoring stress-elicited emissions can provide key insight into immediate stress responses, priming and acclimation. In fact, plant volatiles constitute as yet unexplored, potentially hugely valuable resource for studies on plant stress responses and for rapid phenotyping of plant genotypes according to stress resistance.

The primary goal of this work was to systematically compare bacterial isolates from stressed and mild environments in their capacity to enhance drought stress tolerance of wheat (*Triticum aestivum*). We suggest that bacteria coevolved with plant roots under stress over millennia have a superior potential to enhance drought tolerance of wheat. We further propose a rapid non-invasive method for assessing differences in plant stress tolerance using plant volatiles. To our knowledge, importance of PGPB habitat and involvement of plant volatiles in bacterially-driven drought tolerance enhancement have not been reported before.

## Experimental Procedures

### Bacteria and plant treatment

The bacterial strains from North-Israeli ‘Evolution Canyon’ both from more stressed SFS and less stressed NFS sites (SFS and NFS strains) were isolated as described by us earlier [23]Firmicute *Bacillus thuringiensis* AZP2 was isolated from ponderosa pine (*Pinus ponderosa*) roots grown on gneiss rock at Mt Lemmon, AZ, USA (32.38568° N, 110.69486° W elevation 2150 m) as described by Timmusk et al. [23]. Firmicute *Paenibacillus polymyxa* B was isolated from salty rice (*Oryza sativa*) rhizosphere at Tina plain, Giza, Egypt (31.044° N, 32.6661° E, elevation 13 m) as described earlier [23]. Briefly, the plant roots were carefully shaken and washed in sterile distilled water to remove all loosely attached rock powder and to collect bacteria intimately adhered to plant roots. Plants were placed in sterile plastic bags, transferred to the laboratory, and then stored at +4°C until they were processed in the next day. Plant rhizosphere material (1 g) was homogenized by FastPrep Instrument (BIO 101 Systems), and hence, the rhizosphere macerate contains inner root bacteria. Plant rhizosphere material was suspended in sterile PBS medium (137 mM NaCl, 2.7 mM KCl, 10 mM Na_2_HPO_4_, 2 mM KH_2_PO_4_, pH = 7.4).

The content of endospore-forming bacteria was determined after heat treatment of the soil or plant material suspension at 80°C for 30 min. Tryptic soy agar (TSA) plates were inoculated with 100 µL of heat-treated bacterial suspensions, corresponding to 10^−3^–10^−5^ g soil or plant rhizosphere material per plate. All agar media (pH = 7) contained 15 g agar and 50 mg cycloheximide, to reduce fungal growth. The inoculated Petri dishes were incubated for several weeks at room temperature (∼21°C), and at 30°C, 37°C, or 40°C in boxes together with a beaker of water to keep the agar medium moist.

#### Screening for bacterial metabolic properties and plant drought tolerance enhancement

Bacterial metabolic properties were screened as described by us earlier [23]. Briefly, a small amount of material from 60 bacterial colonies was picked from the TSA plates and transferred to a tryptic soy broth (TSB) medium for a biofilm production assay. The remainder of each colony was streaked on to Dworkin and Foster (DF) plates for the 1-aminocyclopropane-1-carboxylate (ACC) deaminase assay [45]; National Botanical Research Institute's phosphate growth medium (NBRIP) [46] for the P solubilization assay; and high salt medium for halophilicity assay. The TSA plates were used to ensure that some of each colony remained. The endospore-forming ACC deaminase-containing, P-solubilizing and salt tolerant bacteria were retained and studied for their capacity to enhance wheat (*Triticum aestivum*) drought stress tolerance (survival). The colonies that were able to enhance wheat seedling survival were 16S rDNA sequenced from the TSA plate.

Three wheat (*Triticum aestivum*) cultivars (spring wheat cv. Sids1, drought-sensitive winter wheat cv. Stava and drought-tolerant winter wheat cv. Olivin) were used in the present study. Seeds were surface sterilized using 5% chlorine solution. Bacteria were grown in tryptone soy broth (TSB) medium at 28°C overnight. Culture density was determined by colony forming unit analysis (CFU). Inoculation was performed by soaking grains in solutions containing 10^7^ bacteria ml^−1^ for 4 hours at 28°C. For the control treatment, another set of grains was soaked in sterile TSB media. Seeds were sown in plastic pots filled with 450 g sand or sand mixed with 10% greenhouse soil. Both inoculated and non-inoculated treatments were replicated twelve times and each treatment had three plants per pot. The pots were incubated in controlled environment in a MLR-351H (Phanasonic, IL, USA) growth chamber with 24/16°C (day/night) temperature, and 16 h photoperiod at a quantum flux density of 250 µmol m^−2^ s^−1^. The soil moisture was adjusted at 75% of soil water-holding capacity (12.5% of soil dry mass). Soil moisture content was kept constant during the first 10 days of seedling growth.

In 10 days after seed germination, drought stress was induced by stopping watering. Plants grown in sand were stressed for 10 days and plants grown in sand mixed with 10% greenhouse soil were stressed for 14 days. Soil volumetric water content was evaluated using 5TE soil moisture sensors (Decagon Devices, Inc, Pullman, WA, USA). We used 10-days-old wheat plants in our experimental settings as it is well-known that drought tolerance is developmentally regulated and increases with plant age [8]–[10]. plant drought genomics Accordingly, using young plants provides most informative insight into the potential of bacterial priming in increasing plant survival at the most critical stage of plant ontogeny.

### AZP2 identification and quantification

Randomly selected plants were used to confirm that bacteriofilm on AZP2-inoculated plants consisted of AZP2 after 10 days of growth. The roots of selected plants were carefully shaken and washed in sterile distilled water to remove loosely attached soil and to collect bacteria intimately linked to the plant root. Thereafter, the roots were homogenized and the content of endospore-forming bacteria was determined after heat treatment of the soil or plant material suspension at 80°C for 30 min. Tryptic soy agar (TSA) plates were inoculated with 100 mL of these suspensions, corresponding to 10^−3^–10^−5^ g soil or plant rhizosphere material per plate. DNA was isolated from 1-day-old cultures on agar plates. Single colonies were resuspended to obtain a bacterial density of about 10^5^ colony forming units per ml suspension. A 0.3 ml aliquot of the bacterial culture was suspended in 4.7 ml of buffer (10 mM Tris-HCl, pH = 7.6, 50 mM KCl, 0.1% Tween 20). For lysing, the suspension was heated and immediately cooled on ice. The mixture was centrifuged at 6000 g for 5 min and the supernatant was used for PCR analysis. Aliquots of 10 mM of primers 1492R (5′-GGTTACCTTGTTACGACTT-3′) and 27F (5′-AGAGTTTGATCCTGGCTCAG-3′) and 1 ml of template were used. The reaction was performed in 10 ml. The reactions conditions were 95°C for 2 min followed by 30 cycles of denaturation at 95°C for 15 s, annealing at 55°C for 20 s, primer extension at 72°C for 1 min, followed by the final extension at 72°C for 5 min. For sequencing, the PCR products were purified with QIAquickTM Gel Extraction kit (QIAGEN, Hilden, Germany).

### Plant growth analysis

Plant survival was calculated daily after stress application using 32 stressed plants that were randomly selected and divided into two groups with 16 plants each. After stress application, the plants were watered and allowed to recover for 4 days. The recovered plants were counted as survived plants. Eight days after application of drought stress, the survived and recovered plants were harvested, washed and blotted dry between filter paper. Plant roots were counted and their length was estimated with Root Reader3D Imaging and Analysis system [47]. Root-adhering soil was evaluated in twelve plants per treatment. Roots with adhering soil (RAS) were carefully separated from bulk sand and sand soil mix by shaking. Shoot, soil and root dry mass (RT) were recorded after drying the samples at 105°C to a constant mass, and RAS/RT ratio was calculated. Water use efficiency (WUE) was calculated as the ratio of total plant dry mass to total water use during the experiment.

Root hair length and density were evaluated using twelve plants. Plants were carefully separated from soil by shaking. After separation of loosely attached soil, plant roots were washed in distilled water and left to drain in Petri dishes containing 5 ml of water. The other set of plant was homogenised and used for AZP2 identification and quantification. The dried root system characteristics (root hair density and length) were evaluated using Zeiss LSM 710 microscope.

### Protein extraction and antioxidant enzyme activity measurements

Leaf samples for enzyme activity determination were taken after 8 days from drought-treated and well-watered plants. Plant tissue was mixed with 10 ml extraction buffer as described by Knöerzer et al. [48]. The mixture was centrifuged at 14,000 rpm (Eppendorf, 5415C) for 10 min at 5°C, and the supernatant was used to determine protein content and activity of key antioxidant enzymes. Monodehydroascorbate reductase (MDAR) activity was determined following the decrease in light absorbance at 340 nm due to NADH oxidation as described by Hossain et al. [49]. Glutathione reductase (GR) activity was determined by increase in absorbance at 412 nm according to Smith et al. [50]. Superoxide dismutase (SOD) activity was determined by reduction in light absorbance at 490 nm using an Oxiselect SOD activity assay kit (Cell Biolabs, San Diego, CA, U.S.A.) according to manufacturer's instructions. Catalase (CAT) activity was measured by reduction in light absorbance at 520 nm, using an OxiselectTM CAT activity assay kit (Cell Biolabs). For CAT and SOD, enzyme activities were determined per gram of fresh mass (FM). Ultimately, the enzyme activities were normalized with respect to the activities in well-watered plants. The corresponding activities were 20 nmol (mg FM)^−1^ min^−1^ for MDHAR, 25 nmol (mg FM)^−1^ min^−1^ for GR, 0.45 µmol (g FM)^−1^ min^−1^ for SOD and 53 µmol (mg FM)^−1^ for CAT.

### Scanning Electron Microscopy

Environmental scanning electron microscopy (ESEM) micrographs of the samples were obtained with a Hitachi TM-1000- µDex variable pressure scanning electron microscope. Samples were deposited on a carbon tape and coated by gold using Sputter Coater 108 auto (Cressington). Twelve root systems grown in sand soil under water stress were studied for AZP2 biofilm formation.

### Foliage gas exchange measurements

Steady-state net assimilation and transpiration rates and stomatal conductance were recorded immediately after stress application (day 0) and in 2, 5, 8 and 10 days from start of stress application using a Walz GFS-3000 portable gas exchange system equipped with a LED-array/PAM-fluorimeter 3055-FL (H. Walz GmbH, Effeltrich, Germany) [51], [52]. Leaf temperature was set at 25°C, incident quantum flux density at 1000 µmol m^−2^ s^−1^, chamber CO_2_ concentration at 390 µmol mol^−1^ and air humidity at 60%. Multiple leaves were inserted side by side to fill the leaf chamber area. After insertion of leaves, leaves were stabilized under measurement conditions until steady-state gas exchange rates were established, typically 20–30 min before recording the values. After completion of gas-exchange measurements, the leaf chamber fluorimeter was removed and a photograph of the chamber window was taken for enclosed leaf area estimation.

### VOCs sampling and analysis

Volatiles were trapped by sampling 4 L of chamber air from the Walz GFS-3000 cuvette outlet onto a multibed stainless steel cartridge, and analyzed by a Shimadzu 2010Plus GC-MS (Shimadzu Corporation, Kyoto, Japan) as in our previous studies [40], [51], [52]. Ethylene emission rate was measured at days 0, 2, 5, 8 and 10 days after stress application using Picarro G1106 real-time ethylene analyzer (Picarro, Inc, CA, USA). The ethylene analyzer was linked to GFS-3000 gas-exchange system through a bypass loop.

### Data confirmation and validation

Experiments were repeated three times to confirm reproducibility of plant phenotypes. Replicated data were analysed by three-way ANOVA (stress x strain x stress exposure time), and all treatment effects were considered significant at a conservative level of significance of *P≤*0.01.

## Results

### Screening of two sets of rhizosphere bacteria originating from harsh and moderately stressed environments for their drought tolerance enhancement ability

Twelve bacterial isolates from the rhizosphere of wild progenitors of cereals at South Facing Slope (SFS, more stressful site) and twelve isolates from North Facing Slope (NFS, less stressful site) in North-Israeli ‘Evolution Canyon’ isolated and characterized by us earlier were assessed for their capacity to enhance wheat (*Triticum aestivum*, cultivars Stava, Olivin, Sids 1) drought stress tolerance [23]. Briefly, twelve SFS strains were able to grow on ACC as a sole N source, in 2 M NaCl medium and had a high P solubilization index (3–4) and a high biofilm formation value (OD_590_>1). Twelve NFS strains were not able to grow on ACC as a sole N source, neither in 2 M NaCl medium and had a low P solubilization index ≤1.5) and moderate biofilm formation potential (OD_590_≤0.5) [23]. Two to four subpopulations of *Bacillus cereus, B. megaterium, B. pumilus* from SFS and NSF were used for comparison. Only one *Paenibacillus polymyxa* subpopulation was isolated from NSF and was screened along with its four subpopulations from SFS. On average (±SD 16±2% of *P. polymyxa* 15±3%, *B. cereus* 16±2% *B. megaterium* and 10±3%of *B. pumilus* treated plants survived the 10-day drought treatment followed by a four-day recovery period after re-watering. None of the wheat seedlings treated with the same bacterial species representative to NFS survived the drought stress. These results confirmed our hypothesis that harsh environments constitute the source for highly potent rhizosphere bacteria. Therefore, more strains from differently stressed environments were isolated. Two additional isolates from ponderosa pine (*Pinus ponderosa*) rhizosphere at Mt Lemmon AZ, USA, and rice (*Oryza sativa*) rhizosphere at Tina plain, Giza, Egypt were used in this study.

**Figure 1:**
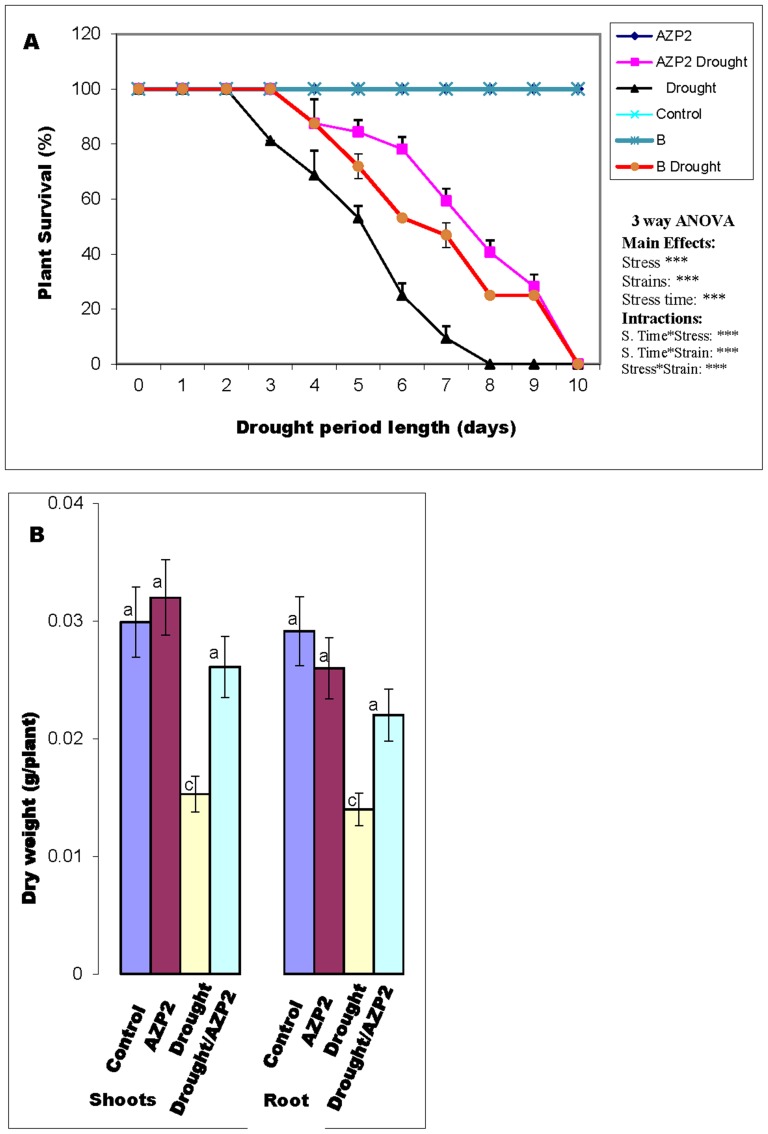
Enhancement of wheat (*Triticum aestivum*) drought tolerance by *Bacillus thuringiensis* AZP2 and *Paenibacillus polymyxa* B in sand soil. Panel A demonstrates the effect of AZP2 and B priming on seedlings survival after a severe 10-day drought stress episode. Panel B shows the effect of AZP2 priming on whole plant dry mass after 8 days growth without watering. The statistical analysis in (A) is based on a three-way ANOVA (stress, strains (i.e. AZP2 and B) and stress exposure time). ANOVA was conducted on two plant groups with 16 replicates in each group. *** indicate highly significant effects for the tested factor at *P≤*0.01. In B, eight independent experiments were performed, and treatments labelled with the same letter are not significantly different at *P≤*0.01.

In comparison to other isolates, *Bacillus thuringiensis* AZP2 isolated from ponderosa pine ecosystem grown under particularly severe nutrient limitation and drought, heat and UV stress exhibited the best potential to enhance plant drought stress tolerance and was chosen for further studies (Table 1). Both the strains *Bacillus thuringiensis* AZP2 and *P. polymyxa* B were able to grow on ACC as the sole N source, 2 M NaCl medium and had high P solubilization index (3.1±0.2). Their biofilm formation value at OD_590_ was 1.0±0.2. On average (±SE), 43±3% of *B. thuringiensis* AZP2- and 23±2% of *P. polymyxa* B-treated wheat seedlings survived the 10-day drought stress. Two additional strains similarly enhanced wheat survival (Fig. 1A, Fig. S2), yet AZP2 priming potential was slightly higher. Hence *B. thuringiensis* AZP2 was the very best strain and this study focuses on AZP2 priming. In addition, *Paenibacillus polymyxa* B strain was used to evaluate the robustness of our method to assess the success of bacterial priming by monitoring alterations in volatile emission and photosynthetic activity.

**Table 1:** Effect of rhizosphere bacterial priming on wheat (*Triticum aestivum L. cv. Stava*) survival under drought stress.

Bacterial strain^1^	Origin	Average (±SD) plant survival (%)^2^
Control		0
*Bacillus thuringiensis AZP2*	Mt. Lemmon, AZ, USA	43±3
*Paenibacillus polymyxa B*	Tina Plain, Giza, Egypt	23±2
*P. polymyxa A26,27, 58,64*	South Facing Slope, ‘Evolution Canyon’SFS,EC) Israel	16±2
*B.cereus A4, 7, 8*	SFS, EC	15±3
*B. megaterium A2, 3, 5*	SFS, EC	16±2
*B. pumilus A1, 3*	SFS, EC	10±3
*P. polymyxa E1*	North Facing Slope, ‘Evolution Canyon’, (NFS,EC) Israel	0
*B.cereus E1, 3, 4, 5*	NFS, EC	0
*B. megaterium E2, 4, 5, 6*	NFS, EC	0
*B. pumilus E3, 5, 6*	NFS, EC	0

1 Patent pending.

2 Plant survival in sand soil after 10-day drought stress followed by 4 days recovery after re-watering.

### Effect of AZP2 on wheat traits and phenotype

The AZP2-inoculated plants were exposed to severe drought stress for 10 days by stopping watering. Cessation of irrigation resulted in strong reductions in soil volumetric water content as evaluated by soil moisture sensors (Fig. S1). During the first two days of stress exposure, no visible signs of drought were recorded. However, after the third day, plant survivorship decreased significantly, and this trend was reinforced with increasing the stress period. Less than 20% of non-inoculated plants could survive for seven days of stress, and none could survive for eight days of drought (Fig. 1A).

In contrast, AZP2-inoculated plants exhibited a delayed initial response to drought stress. After six days of drought stress, 80% of *B. thuringiensis* AZP2-primed plants survived and more than 20% of *B. thuringiensis* AZP2-primed plants survived after nine days of stress (Fig. 1A). All AZP2-primed plants were severely wilted after 10-day water stress (Fig. 1A). However, after 4 days of recovery treatment (re-watered to soil field capacity, i.e. 12.5% of soil dry mass), 43% of primed seedlings were able to recover, while none of the non-primed seedlings recovered (Table 1). Root and shoot dry mass of both control and drought-stressed plants were significantly increased in the case of AZP2 priming (Fig. 1B). Taken together, the bacterial priming resulted in 100% seedling germination, 78% increase in plant dry mass and five times greater average plant survival grown under severe drought and nutritional stress.

**Figure 2:**
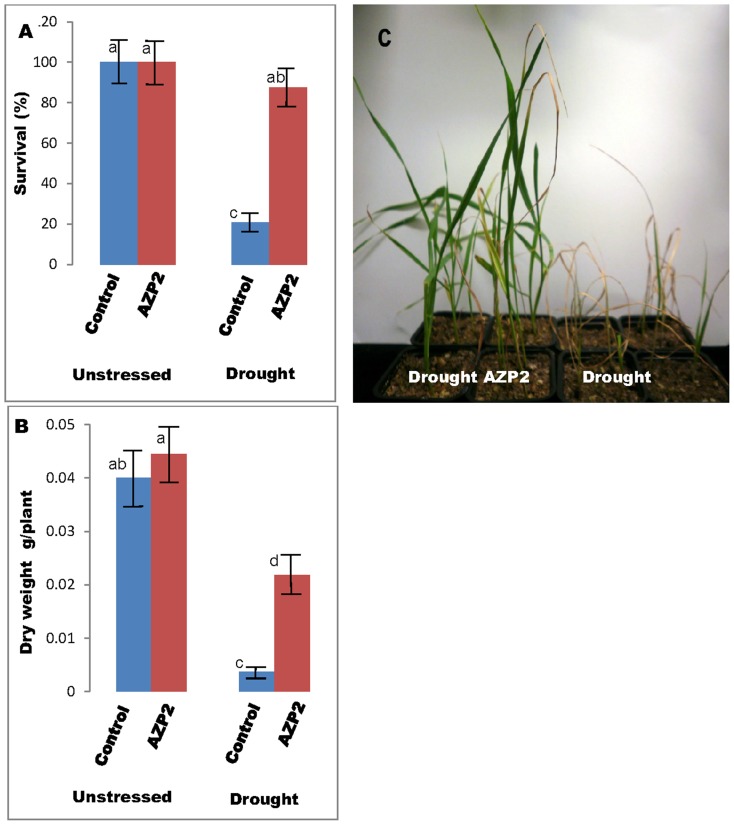
Increase of wheat drought stress tolerance by *Bacillus thuringiensis* AZP2 in sand mixed with 10% greenhouse soil. Effect of AZP2 priming on wheat survival (A, C) and dry mass (B) after 14 days of drought stress. Eight independent experiments were performed, and treatments with the same letter are not significantly different at *P≤*0.01.

Slightly improved soil nutrition further improved the beneficial effects of AZP2 priming. Growing wheat in sand mixed with 10% greenhouse soil and drought-stressed for 14 days resulted in major phenotypic differences (Fig. 2A–C). The non-primed plants were severely damaged after such acute water stress, but *B. thuringiensis* AZP2 priming strongly reduced the drought-induced damage, and increased plant survival and final dry mass (Fig. 2A–C). While about 43% of AZP2 plants survived in sand after 10 days of drought stress followed by 4 days recovery after re-watering, 89% survived in sand with greenhouse soil after 14 days of drought stress followed by 4 days recovery (Table 1 and Fig. 2A–C).

Detailed analysis of AZP2-primed wheat seedlings revealed that AZP2 drought tolerance improvement was always associated with longer and denser root hair (Table 2). AZP2 effect on root hair formation was observed under normal watering regime, and this trait was gradually more expressed with increasing the length of the drought stress treatment (Table 2). Changed root hairiness was the major morphological difference in comparison to non-inoculated plants observed during first five days of drought stress both in sand as well as in sand supplemented with 10% greenhouse soil. Furthermore, AZP2 reduced root length under watering, but significantly increased density and length of lateral roots under drought stress in sand soil (Table 2). Compared to non-primed controls, two to three times more lateral roots were observed in AZP2-primed plants (Table 2).

**Table 2:** Effect of priming by *Bacillus thuringiensis* AZP2 on wheat *(Triticum aestivum L. cv. Stava)* on average (±SD) growth characteristics, water use efficiency and antioxidant enzyme activities.

	Well-watered		Drought-stressed	
	Control	AZP2-primed	Control	AZP2-primed
Average plant survival improvement (%)				**500**
Plant dry mass increase (%)		9		78
Germination rate (%)	72	**100**	50	**100**
Lateral root count^1^	266±76^ab^ *	235±21^ab^	181±16^c^	**192.5±3.5^bc^**
Total lateral root length (cm)	263±11^a^	183±23^b^	89±11^d^	**142±31^bc^**
Longest root length (cm)	28.4±1.4^ab^	27.6±2.2^ab^	20.7±1.2^c^	23.2±1.2^bc^
Soil attached to root^2^ (g g^−1^ dry root)	**62±19^a^**	**92±30^ab^**	**10±5^c^**	**25±11^bc^**
Average root hair length^3^ (mm)	**0.74±0.21^a^**	**1.5±0.5^b^**	**0.84±0.21^a^**	**1.91±0.22^b^**
Root hair density (number per mm^3^)	**24.1±3.1^a^**	**30.1±2.0^ab^**	**24.1±2.0^a^**	**32.0±1.1^b^**
Number of fresh roots per plant	nt	nt	**3.0±1.1^a^**	**10.1±2.0^b^**
Water use efficiency^4^ (g g^−1^)	0.1053±0.0031^d^	0.1087±0.0021^d^	**0.0802±0.0032^b^**	**0.135±0.012^c^**
Relative Enzyme Activities:^5^				
MDHAR	1±0.1^a^	1.4±0.2^a^	**1.8±0.1^b^**	**2.6±0.2^c^**
GR	1±0.2^a^	1.3±0.1^a^	**1.4±0.3^a^**	**1.8±0.2^c^**
SOD	1±0.2^a^	1.3±0.1^ab^	**0.7±0.1^ab^**	**1.9±0.2^c^**
CAT	1±0.1^a^	1±0.2^a^	**1.5±0.1^bc^**	**2±0.2^c^**

1 Analysis of plant root was conducted by Root Reader3D Imaging and Analysis System and manually [7].

2 Twelve plants per treatment were sampled. Roots with adhering soil (RAS) were carefully separated from bulk soil by shaking. Soil and root dry mass (RT) was recorded after drying the samples at 105°C, and RAS/RT ratio was calculated.

3 Twelve plants were carefully separated from soil by shaking followed by washing the roots in distilled water and left to drain in Petri dishes with water to maintain humidity. Root system characteristics were evaluated by Zeiss LSM 710 microscope.

4 Water use efficiency is defined as the ratio of total plant dry mass per total water used.

5 MDHAR - Monodehydroascorbate reductase, GR- Glutathione reductase, SOD- Superoxide dismutase, CAT-Catalase.

See Materials and Methods for enzyme extraction and activity measurements.

*Means followed by the same letter are not significantly different at p≤0.01. See Experimental procedures.

**Figure 3:**
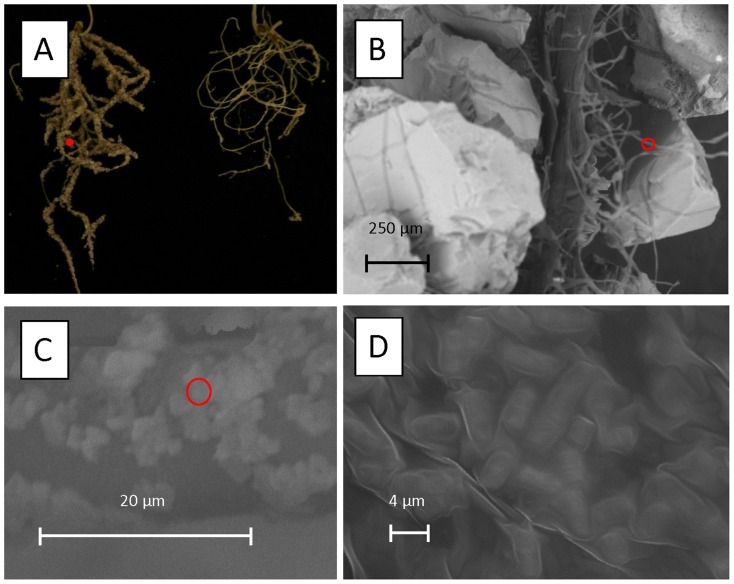
Formation of sand soil mulch and biofilm on root hairs of winter wheat (cv. Stava) by *Bacillus thuringiensis* AZP2. Scanning electron micrographs were made of AZP2-primed wheat root systems after 10-day drought stress and show sand mulch (A, B) and bacterial biofilm formation on root hair (C, D). Significantly more soil mulch is attached to the AZP2 treated plant (A, right) in comparison to control (A, left). Red circles indicate the areas magnified.

Bacterial biofilm formation on plant root surface was estimated as the amount of soil attached to roots. Two to three times more soil was attached to AZP2-primed roots under drought stress and up to two times more under normal watering (Table 2). Electron microscopic imaging of the AZP2-primed wheat seedlings grown under drought stress confirmed the bacterial biofilm formation on root hairs (Fig. 3). Overall, in AZP2-primed plants, water use efficiency increased by 63% in comparison to non-primed control plants.

### Plant photosynthetic activity is improved by the drought tolerance enhancing bacteria

**Figure 4:**
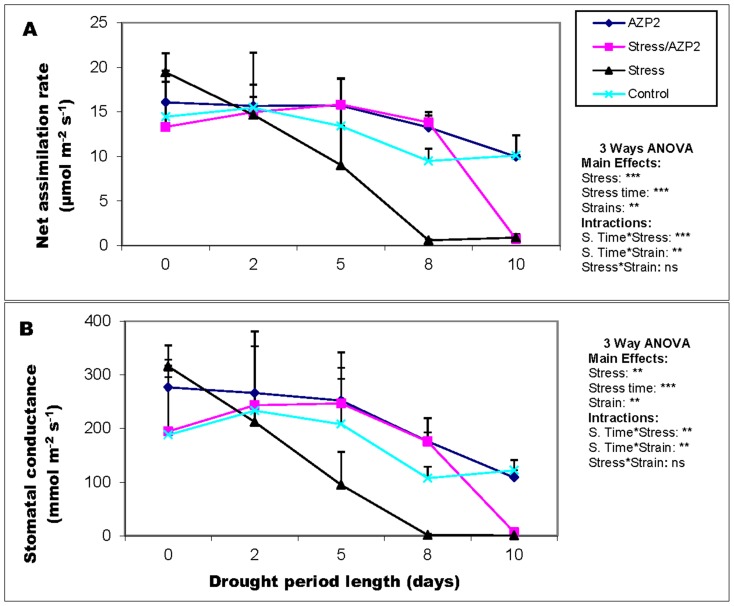
Net assimilation rate (A) and stomatal conductance (B) of *Bacillus thuringiensis* AZP2-primed wheat seedlings under drought stress. The data are shown for plants grown for 0, 2, 5, 8 and 10 days without water. The error bars indicate +SE for three biological replicates. Statistical analysis is based on three-way ANOVA with stress, strains (*Bacillus thuringiensis* AZP2 vs. *P. polymyxa* B) and stress exposure time as factors. ***, ** and ns, indicate highly significant, significant or non-significant effects for the tested factor at *P<*0.05.

Foliage net assimilation rate and stomatal conductance were monitored through the drought stress treatment and in well-watered control treatment in both primed and non-primed plants. Under drought-stress, a steady decline in net assimilation rate was recorded in all stressed wheat plants (Fig. 4A). Net assimilation rate was almost totally inhibited in non-primed wheat plants in 8 days since withholding water. This result was qualitatively the same in plants inoculated with *P. polymyxa* E1 and in plants inoculated with *B. cereus* E1 from NFS. (Fig. 4A). However, *B. thuringiensis* AZP2-primed plants exhibited much higher net assimilation rate compared to the non-inoculated controls, independently of whether the plants were stressed or not. The only exception was the 10-day drought-stressed treatment in primed plants where the net assimilation rate was almost completely inhibited. No significant difference in net assimilation rates among *B. thuringiensis* AZP2- and *P. polymyxa* B-primed plants was observed. A regression analysis demonstrated a very strong positive correlation between net assimilation rate and plant survivorship through the drought-stress period (*r* ^2^ = 0.95, *P*<0.001; Fig. S2A).

Stress- and priming-driven modifications in stomatal conductance (*g*_s_) mirrored those in net assimilation rate. Non-primed control treatment showed relatively stable *g*_s_ between 150 and 200 mmol m^−2^ s^−1^ throughout the course of the experiment (Fig. 4B). In contrast, drought-stressed non-primed plants showed a steady reduction in *g*_s_ during the drought treatment. *Bacillus thuringiensis* AZP2-primed plants had significantly higher *g*_s_ whether drought-stressed or not (Fig. 4B). Changes in stomatal conductance during the drought cycle were positively correlated with plant survivorship (*r* ^2^ = 0.91, *P*<0.001; Fig. S2B).

### AZP2-colonization upregulates the ascorbate-glutathione cycle, CAT and SOD

Due to the importance of antioxidant enzymes in ROS scavenging, the activities of monodehydroascorbate reductase (MDHAR), glutathione reductase (GR), catalase (CAT) and superoxide dismutase (SOD) were studied after 8 days in drought-stressed and well-watered plants. Enzyme activities were normalized to those of well-watered seedlings. The relative activity of MDHAR was affected increased by water stress and AZP2 colonization (Table 2). Activity of GR was slightly enhanced by drought, and strongly enhanced by AZP2. Both SOD and CAT activities were increased by AZP2 under drought stress (Table 2).

### Benzaldehyde, geranyl acetone and β-pinene show distinct patterns in response to bacterially induced plant drought stress tolerance

The GC-MS analysis showed that seven terpenoid and benzenoid compounds were emitted from wheat leaves including α-pinene, limonene, *para*-cymene, α-phellandrene and camphene. Among the compounds, benzaldehyde, β-pinene and geranyl acetone were most responsive to drought stress and exhibited greatest differences among the treatments, while some other compounds such as ethylene emission rate were similar among the treatments (data not shown).

**Figure 5:**
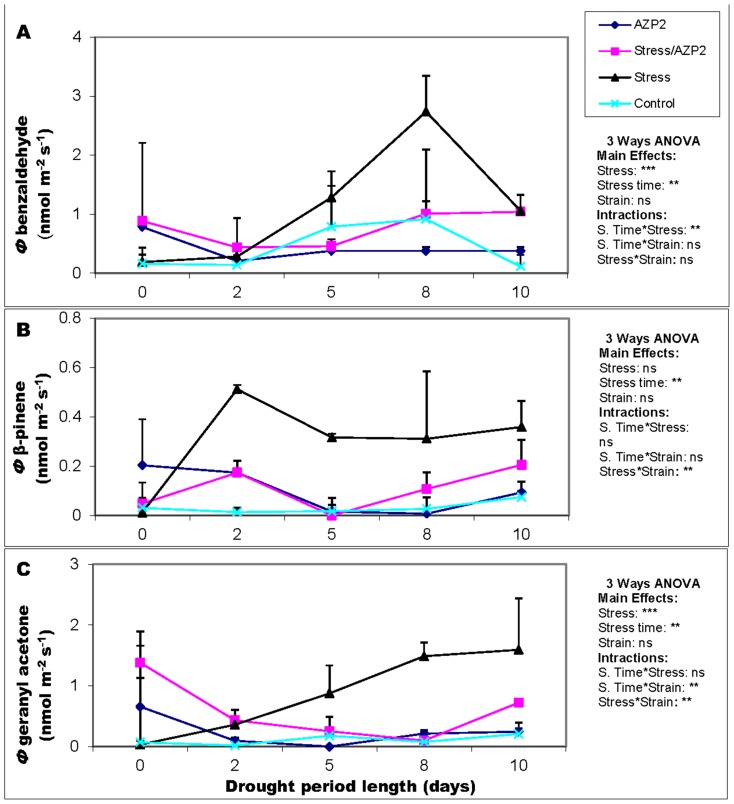
Temporal variations in the emission rates of some benzenoids and terpenoids emitted by wheat plants. Benzaldehyde (A), β-pinene (B) and geranyl acetone (C) emission rates from leaves of drought-stressed (0, 2, 5, 8 and 10 days without water) wheat plants after priming with *Bacillus thuringiensis* AZP2 are demonstrated. The error bars indicate +SE for three biological replicates. Statistical analysis and levels of significance as in Fig. 4.

Benzaldehyde emissions increased with increasing the drought stress period. The emission reached a maximum value when non-primed wheat plants were grown without water for 8 days (Fig. 5A). On the other hand, *B. thuringiensis* AZP2-primed stressed plants showed modest benzaldehyde emission compared to the non-primed stressed plants. Both *B. thuringiensis* AZP2- and *P. polymyxa B*-primed stressed plants showed similar benzaldehyde emission patterns and the benzaldehyde emission rate was negatively correlated with plant survival under stress conditions (*r* ^2^ = 0.96, Fig. S3A and Fig. 5, three-way ANOVA).

Low-level β-pinene emissions were detected from well-watered wheat plants independent of whether the plants were primed or not. However, two-fold higher β-pinene emissions were detected already in two days after drought stress, and the emissions further stabilized to a somewhat lower steady level for the rest of the study. *B. thuringiensis* AZP2-primed stressed plants kept their β-pinene emissions at a low level similar to the non-stressed plants (Fig. 5B).

Drought stress induced a steady increase in geranyl acetone emission (Fig. 5C). *Bacillus thuringiensis* AZP2-primed stressed plants maintained a significantly lower geranyl acetone emission compared with their non-primed counterparts. Strong negative correlations were observed between geranyl acetone emissions and plant survivorship (*r* ^2^ = 0.97, *P*<0.001) and net assimilation rate (*r* ^2^ = 0.994, *P*<0.001) (Figs.S3B and C).

Among the compounds, benzaldehyde, β-pinene and geranyl acetone showed significant differences in response to drought stress in wheat plants primed with different strains. Compared with *P. polymyxa B*-primed plants, in plants primed with *Bacillus thuringiensis* AZP2 these compounds were elicited to a lower degree under the most severe drought stress of 6–8 days since withholding irrigation water (Fig. 5 and SIFig.3). In wheat inoculated with *P. polymyxa* E1 and *B. cereus* E1 from NFS no differences in the degree of elicitation of volatile emissions compared with controls were observed (data not shown).

## Discussion

### Rhizospheric bacteria from harsh environments induce drought stress tolerance in modern wheat

Achieving high yields of crop plants under global irrigation water shortage is the main challenge for agriculture. Microorganisms could play a significant role in providing viable solutions, but we first must understand and exploit their unique properties and develop methods for monitoring their performance [11], [14], [16]–[19], [53].

The first report on enhancement of plant drought stress tolerance by rhizosphere bacteria has been published in 1999, and several *Paenibacillus* sp. and *Bacillus sp*. and some other gram-positive bacterial isolates were shown to be effective in improving plant drought stress tolerance (8). In our study, we observed five times greater survival and 78% higher biomass in inoculated plants under drought stress (Table 1), confirming the potential of bacterial priming in enhancing plant performance under drought. To the best of our knowledge, such a strong improvement of drought tolerance by bacterial inoculation has not been reported before.

Until now, there has been no studies investigating the importance of the habitat of free-living plant growth-promoting bacteria (PGPB) on their capacity to affect plant drought tolerance. *Bacillus thuringiensis* AZP2 strain was isolated as a ponderosa pine root endophyte from highly stressed environment in Arizona, USA. The tree seedlings had been growing on gneiss rock under UV, heat and drought stress and severe nutritional deprivation. In our study, 43% of *B. thuringiensis* AZP2 treated wheat plants survived the severe 10-day drought stress exposure followed by 4 days recovery after re-watering (Table 1). The second best isolate, *Paenibacillus polymyxa B* was isolated from Tina plain, Giza, Egypt, where rice plants had been growing under severe salt stress over prolonged period. In our study, 23% of *P. polymyxa B* treated wheat plants survived the 10-day drought stress.

Our results further revealed that 12 bacterial subpopulations from harsh SFS environments in ‘Evolution Canyon’ in Israel had much greater potential to alter drought resistance of wheat than 12 subpopulations from more moderate NFS environments (Table 1). 16% of *P. polymyxa*, 15% of *B. cereus*, 16% of *B. megaterium* and 10% of *B. pumilus* treated plants from SFS survived the drought stress (Table 1). In contrast, the isolates of the same species from moderate NFS did not significantly affect wheat drought tolerance (Table 1) [23].

All drought tolerance-enhancing bacteria tested were biofilm-forming, ACC deaminase-containing and had a high P-solubilizing activity. These metabolic qualities have likely contributed to their survival in harsh environments, and are likely responsible for their capacity to increase plant drought tolerance [23]. We argue that rhizospheric bacteria have coevolved with their host plant roots under stressful conditions, leading to fine-tuned regulatory gene expression networks at multiple levels to respond with high flexibility to diverse stresses in the belowground environment. Taken together, our results suggest that the bacteria from harsh environments are more likely to contribute plant stress tolerance enhancement.

### Enhancement of plant drought tolerance by rhizosphere bacteria depends on dynamic interactions among bacteria, roots, soil, and water in the rhizosphere

Despite several mechanisms suggested, the mode of how rhizosphere bacteria enhance plant drought stress tolerance is largely unknown. In particular, we need to ask how *B. thuringiensis* AZP2 priming enhances wheat drought tolerance. *Bacillus thuringiensis* AZP2 genome has been sequenced using Ion Torrent PGMTM system (unpublished data of Salme Timmusk). Comparative genomic analysis of the sequence reveals gene clusters for alginate, ACC deaminase, and auxin (IAA) production and regulation. Any of the traits alone, and in combination could have been responsible for the bacterial drought tolerance enhancement [22], [54], [55].

Our results indicate that dynamic interactions among bacteria, roots, soil, and water in the rhizosphere determine what mechanism is responsible for the bacterial-induced wheat drought tolerance enhancement at specific stages of seedling growth Table 2 and [56]. Plant seedling successful establishment is required for its survival, and hence, AZP2-induced high germination rate is of prime importance at this stage of development. In fact, AZP2 inoculation resulted in 100% germination of wheat seeds (Table 2).

Following germination, the capacity of roots to extract moisture and nutrients from the soil become the key traits determining plant survival. Improved nutrient and water extraction capacity can be achieved by various ways. Our results indicate that AZP2 inoculation resulted in two to three times longer root hairs, and longer and denser lateral roots (Table 2). These effects were even more pronounced under longer drought stress periods [56]. Root hair length and density are critical when it comes to water and nutrient acquisition from surrounding environment. Although root hair formation can be massively enhanced, this increase should not necessarily show up as an increase in total root dry mass. Hence our results also draw attention to a general misinterpretation of the importance of ‘root mass’ as a criterion in showing the effect of inoculation. Based on our results, root hair microscopic observation are highly recommended in parallel of studying total root mass.

In our experiments, especially in the sand, plants were exposed to both nutrient limitation and drought stress. Under any abiotic stress, there is a significant decrease in photosynthesis and, consequently, a reduction in the amount of metabolites and energy. It is important for plants to use this reduced amount of resources to maximize their growth and reproductive potential. Therefore, under particularly severe nutrient starvation in sand soil, increased proliferation of lateral roots was observed (Table 2). This contributes to enhanced exploitation of the topsoil where bioavailable nutrients are typically located in the field relative to the subsoil which is depleted of nutrients. On the other hand, under drought stress, lateral root growth inhibition and enhancement of vertical root growth are usually adaptive responses, because water availability is usually higher in deeper soil layers. Thus, restriction of lateral root growth in the top-layer of sand soil supplemented with more fertile greenhouse soil can be an adaptive modification contributing to greater drought tolerance of wheat. Far more genes than generally believed (more than 300) have been found to be involved in lateral root development [57], and clearly more experimental work is needed to gain insight into regulation of lateral root development as driven by soil nutrient and water availabilities.

Another important root trait in plant protection against drought stress is the creation of bacterial biofilm with attached soil mulch. AZP2-induced denser and longer root hair framework forms an excellent matrix for the bacterially-excreted biofilm comprised of cells and extracellular matrix producing a thick sticky layer around root hair (Fig. 3). Hence, induction of long and dense root hair should be considered as an important drought stress tolerance enhancement strategy. The dense biofilm matrix also limits diffusion of biologically active compounds secreted by bacteria and these are therefore concentrated on the root surface, facilitating plant uptake. In addition, biofilm formation on root hair substantially improves root-to-soil contact, enhancing plant nutrient acquisition from soil and suggesting that biofilm formation importantly contributes to improving plant nutrition as well (Table 2).

Alginate, a hygroscopic bacterial polysaccharide, can play an important role in determining the biofilm capacity to enhance water status of seedlings [58]. Bacterial alginate water-holding capacity is very high and it loses water slowly [58]–[60], thereby keeping root cells hydrated long enough to allow for cellular metabolic adjustments necessary to enhance drought stress tolerance. The drought tolerance enhancement of alginate might be due to its hygroscopic properties, but can also result from its role in biofilm architecture that contributes to reduced evaporation loss [58]. We have shown that small amounts of alginate are produced by AZP2 under controlled conditions (manuscript in preparation), and thus, the alginate mechanism in AZP2-induced drought tolerance is plausible.

Another important trait for plant successful establishment under stressful conditions is regulation of root development. After 10 days of drought stress, untreated plant root systems were composed of dry thin roots with very few fresh vital roots (Table 2). Three times more fresh lateral roots were counted on AZP2-primed wheat seedlings. This is in agreement with our earlier suggestion that the biofilm of rhizosphere bacteria together with soil mulch has protective role against drought stress [17], [61]. Yet the results also indicate production of biologically active compounds for root maturity regulation in the bacterial biofilm.

Taken together, AZP2 inoculation increases lateral root density and length, and the inoculation enhanced even more strongly root hair density and length (59% and 200% respectively). We conclude that both lateral root and root hair increase in drought-stressed AZP2-inoculated wheat have likely resulted from bacterial production of IAA and ACC deaminase, and perhaps from additional chemical modifications [56]. Any bacterially-secreted compound required for plant drought stress tolerance enhancement is kept in place inside the bacterially-formed and root hair-supported alginate-containing biofilm. The compounds are thus likely to be efficiently taken up by the plant and result in elicitation of gene expression networks leading to more stress-adapted wheat phenotypes (Table 2, Fig. 3) [56].

### Gauging volatiles provides an effectual platform for rapid screening of potent bacterial strains

Our results show that ROS scavenging enzyme activities increase in response to bacterial priming, especially under drought stress (Table 2). Similar alterations in antioxidative capacity by stress-alleviating microorganisms have been reported earlier [32], [33]. In principle, ROS detoxification capacity could be used as a parameter in screening for drought stress-alleviating potential of microorganisms. Any of rapid and effective screening methods should detect plant stress in early phases before the stress becomes that severe that it can be detected by visual symptoms. Such an early monitoring gives the possibility to cope with the stress situation in its early stage before irreversible damage occurs and the yield of crop plants is seriously reduced. Yet, the major shortcoming of many stress detection methods, including ROS detoxification capacity, is that they are invasive [7] for a review, i.e. to measure plant stress status, the plant has to be entirely or partly harvested and processed. We have aimed to contributing towards development of a non-invasive strategy to screen plant stress and its possible alleviation by potent bacterial strains.

Different volatile organic compounds (VOC) are commonly emitted from plants leaves and these emissions are known to increase substantially under stress situations [35], [41]–[43], [52], [62]. Some of these VOCs are by-products of stress-elicitation of various physiological processes, while others are used as sophisticated signals by which plants communicate stress within and among the plants and that trigger stress tolerance in receiver organs and plants situated farther from the stress source [63]–[65]. Although volatile emissions result in significant carbon losses and can lead to plant growth penalties in non-stressed situations [66]–[68], many of the stress-released volatiles play important physiological and ecological roles under stress.

We have previously demonstrated that abiotic stress can lead to elicitation of monoterpene emissions from wheat, and the emissions were quantitatively correlated with the severity of stress [42], [43]. Our results clearly showed that the elevated emission of VOCs was always negatively correlated with plant growth and fitness under drought stress conditions. It has been demonstrated that plants may lose up to 10%, (exceptionally up to 50%), of the carbon fixed by photosynthesis as cost for VOCs emission under stressful conditions [69]. In addition to typical stress volatile hormones such as ethylene, stresses result in upregulation of several secondary metabolic pathways including terpenoid and shikimic acid pathway [70], and the observation of enhanced emission of certain terpenoids and benzenoids in stress is in agreement with upregulation of these key secondary metabolic pathways.

Overall, the emission rates of induced VOCs are quantitatively associated with the severity of the stress [41], [71], [72]. Here we further observed that the emission rates of key stress-elicited volatiles increased with increasing the severity of drought stress (Fig. S3). The observation that VOC emissions were significantly reduced and were correlated with higher photosynthesis and plant survival under drought stress in plants primed by rhizosphere bacteria suggests that bacterial inoculation improved plant stress tolerance. Reduced emissions of stress-induced VOCs further imply lower cost for VOC emission, potentially contributing to greater productivity under stress. Volatile detectors capable of detecting very small amounts of volatiles in outdoor conditions are already commercially available, although the cost is still quite high e.g. [73,74–76]. Hence, monitoring of drought stress development and its potential alleviation by rhizosphere bacteria by VOC in realistic biological settings in field might be feasible in the near future. Such measurements could be conducted in parallel with quantitative PCR screening of bacterial strains involved, and quantification of bacterial bioactive compound production.

## Conclusions

Certainly, more bacterial strains should be tested in the future to gain insight into the sensitivity of the suggested method. In particular, in discriminating among different bacterial strains at different stages of plant development. Yet, our results demonstrate that plant inoculation with bacteria from harsh environments resulted in significantly higher survival of drought-stressed plants, and in greater photosynthesis and biomass production. This was reflected in modifications in volatile profiles and total emission rates, consistent with the overall hypothesis that measurements of volatiles can provide a promising novel methodology to gauge stress resistance. The results collectively point out that inoculation with bacteria from harsh environments could be the way for rapid enhancement of plant stress tolerance. We suggest that monitoring of the elicitation of VOC emissions is a promising method to characterize the efficiency of different bacterial strains in priming for drought stress resistance.

## Supporting Information

Figure S1 **Volumetric water content of the rhizosphere of** ***Bacillus thuringiensis*** **AZP2- and** ***P. polymyxa*** **B-primed and non-primed wheat seedlings under watering and after 10 days of drought exposure.**
(TIF)

Figure S2 **Correlations of plant survival with net assimilation rate (A), and with stomatal conductance (B) in wheat seedlings drought-stressed for 0, 2, 5, 8 and 10 days.** The correlations are significant at *P*<0.01.
(TIF)

Figure S3 **Plant survival in relation to emission rates of benzaldehyde (A), and geranyl acetone (B) and geranyl acetone emission rate in relation net assimilation rate (C) on wheat seedlings drought-stressed for 0, 2, 5, 8 and 10 days.** The relationships are significant at *P*<0.01.
(TIF)
