# Supplementary figures and images for: Drought-Tolerance of Wheat Improved by Rhizosphere Bacteria from Harsh Environments: Enhanced Biomass Production and Reduced Emissions of Stress Volatiles
Source: PLoS One. 2014 May 8;9(5):e96086. doi: 10.1371/journal.pone.0096086 (PMC4014485; doi:10.1371/journal.pone.0096086)

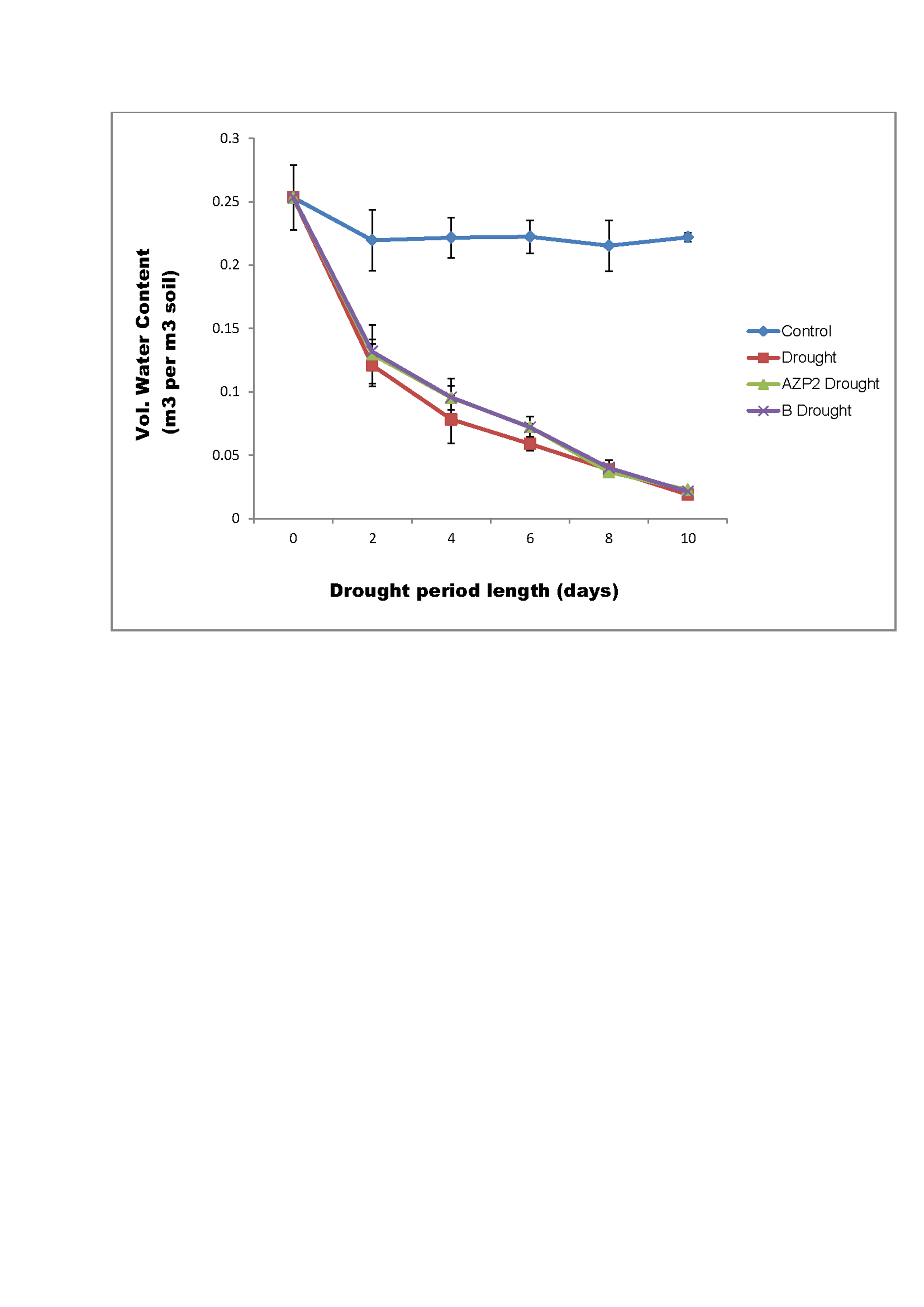

Supplement: Figure S1 — Volumetric water content of the rhizosphere of Bacillus thuringiensis AZP2- and P. polymyxa B-primed and non-primed wheat seedlings under watering and after 10 days of drought exposure. (TIF) [file pone.0096086.s001.tif]

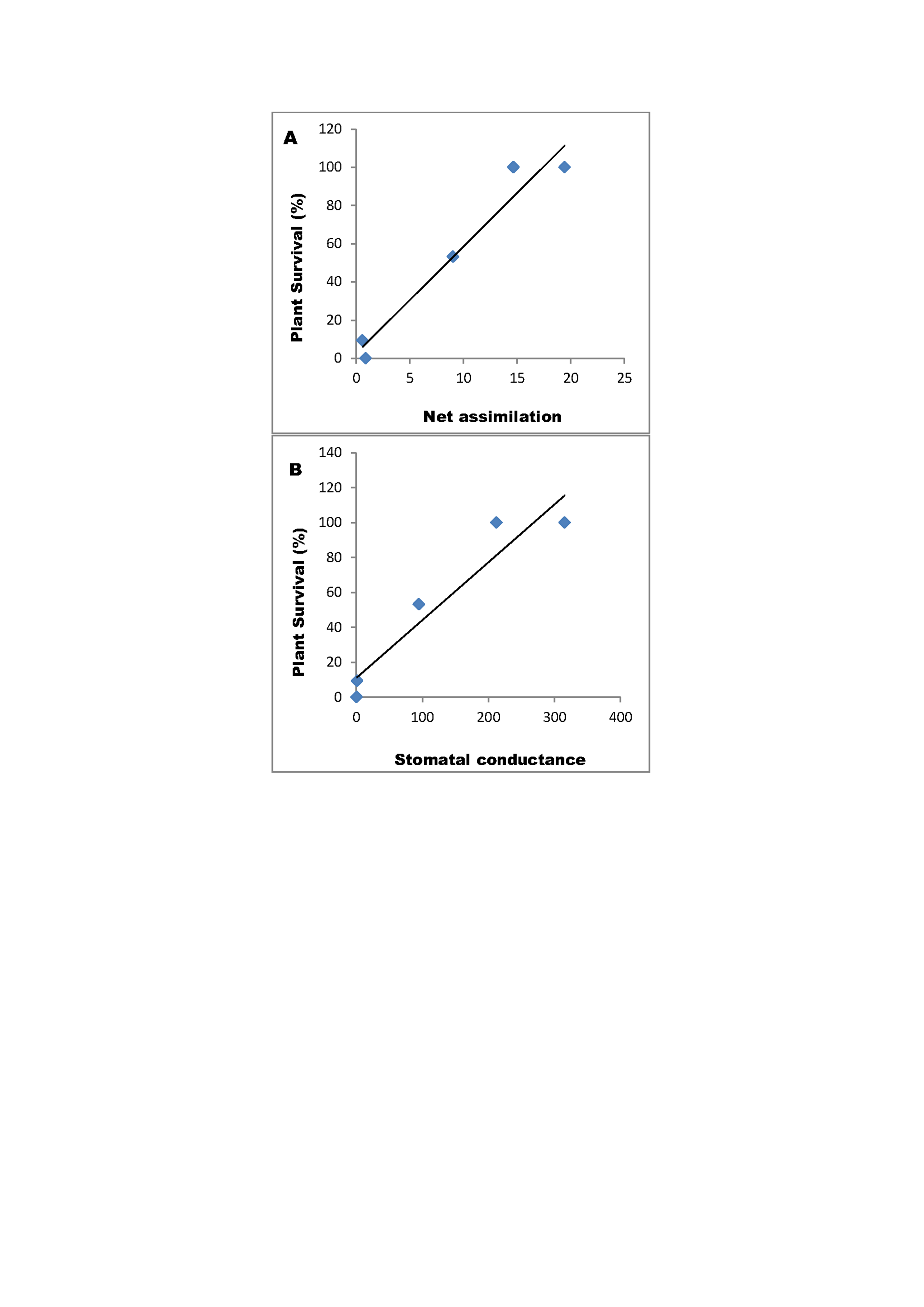

Supplement: Figure S2 — Correlations of plant survival with net assimilation rate (A), and with stomatal conductance (B) in wheat seedlings drought-stressed for 0, 2, 5, 8 and 10 days. The correlations are significant at P<0.01. (TIF) [file pone.0096086.s002.tif]

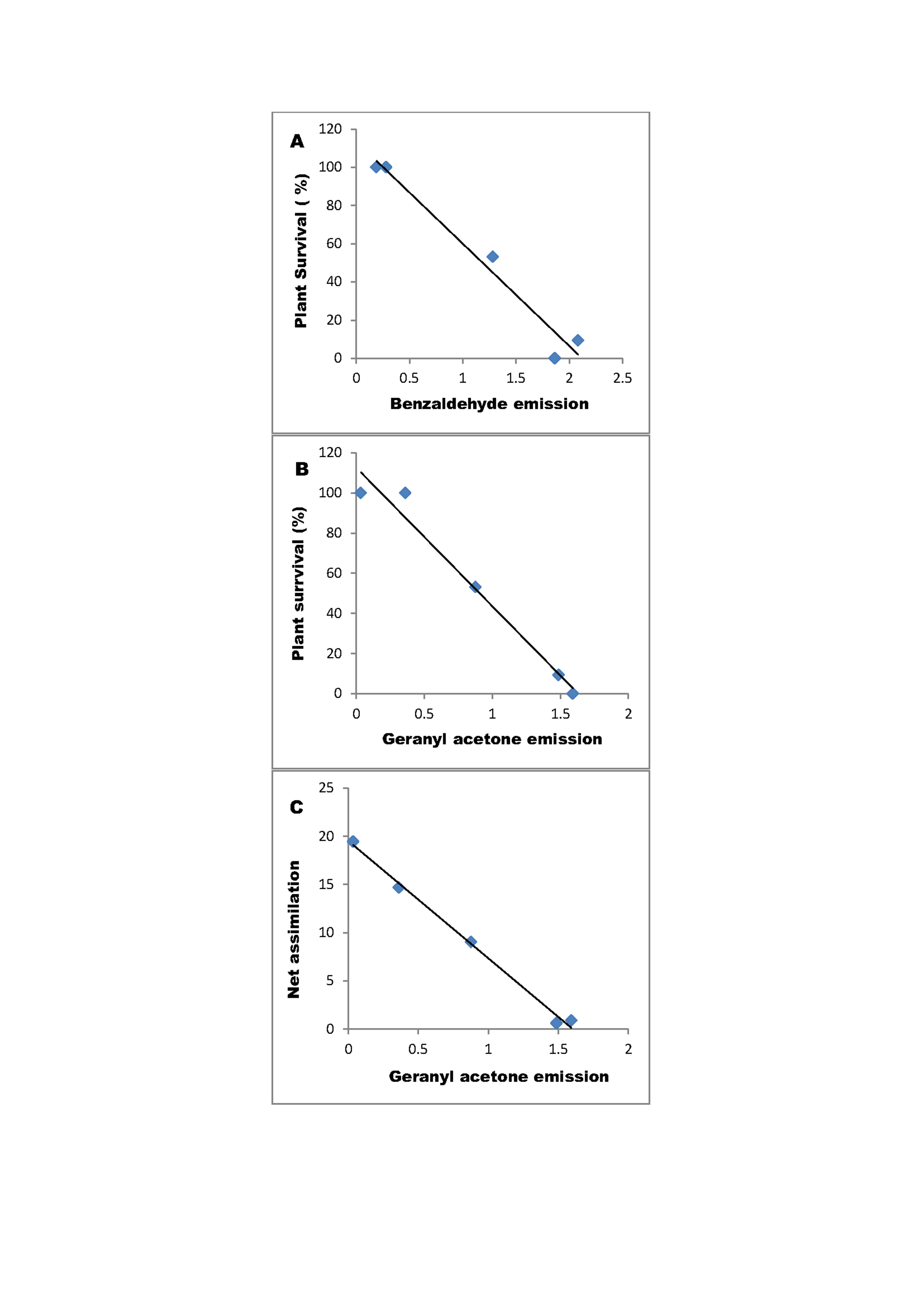

Supplement: Figure S3 — Plant survival in relation to emission rates of benzaldehyde (A), and geranyl acetone (B) and geranyl acetone emission rate in relation net assimilation rate (C) on wheat seedlings drought-stressed for 0, 2, 5, 8 and 10 days. The relationships are significant at P<0.01. (TIF) [file pone.0096086.s003.tif]
